# Understanding Healthcare Students’ Experiences of Racial Bias: A Narrative Review of the Role of Implicit Bias and Potential Interventions in Educational Settings

**DOI:** 10.3390/ijerph182312771

**Published:** 2021-12-03

**Authors:** Olivia Rochelle Joseph, Stuart W. Flint, Rianna Raymond-Williams, Rossby Awadzi, Judith Johnson

**Affiliations:** 1 School of Psychology, University of Leeds, Leeds LS2 9JU, UK; S.W.Flint@leeds.ac.uk (S.W.F.); j.johnson@leeds.ac.uk (J.J.); 2 Bradford Institute for Health Research, Bradford Royal Infirmary, Temple Bank House, Duckworth Lane, Bradford BD9 6RJ, UK; 3 Scaled Insights, Nexus, University of Leeds, Leeds LS2 3AA, UK; 4 School of Health and Life Sciences, Glasgow Caledonian University, London E1 6PX, UK; Rianna.RaymondWilliams@gcu.ac.uk; 5 Postgraduate Graduate Medical Education, Northwick Park Hospital, London HA1 3UJ, UK; R.awadzi@nhs.net; 6 School of Public Health and Community Medicine, University of New South Wales, Sydney 2052, Australia

**Keywords:** healthcare placement, healthcare education, implicit racial bias, diversity

## Abstract

Implicit racial bias is a persistent and pervasive challenge within healthcare education and training settings. A recent systematic review reported that 84% of included studies (31 out of 37) showed evidence of slight to strong pro-white or light skin tone bias amongst healthcare students and professionals. However, there remains a need to improve understanding about its impact on healthcare students and how they can be better supported. This narrative review provides an overview of current evidence regarding the role of implicit racial bias within healthcare education, considering trends, factors that contribute to bias, and possible interventions. Current evidence suggests that biases held by students remain consistent and may increase during healthcare education. Sources that contribute to the formation and maintenance of implicit racial bias include peers, educators, the curriculum, and placements within healthcare settings. Experiences of implicit racial bias can lead to psychosomatic symptoms, high attrition rates, and reduced diversity within the healthcare workforce. Interventions to address implicit racial bias include an organizational commitment to reducing bias in hiring, retention, and promotion processes, and by addressing misrepresentation of race in the curriculum. We conclude that future research should identify, discuss, and critically reflect on how implicit racial biases are enacted and sustained through the hidden curriculum and can have detrimental consequences for racial and ethnic minority healthcare students.

## 1. Introduction

Despite increasing diversity in the general population of high-income countries, empirical evidence of differential attainment, higher attrition [1,2,3], and negative experiences in healthcare students from racial and ethnic minority groups continues to persist [4,5,6]. Furthermore, efforts to increase the racial and ethnic diversity of students within healthcare education to be in line with local populations have shown slow progress [7,8,9]. For example, a cross-sectional study utilized the Association of American Medical Colleges (AAMC) and US Census data to examine self-reported race and ethnicity of US medical school applicants and matriculates between 2002–2017 in relation to the general population [8]. Lett et al. [8] reported that Black, Hispanic, Asian, American Indian or Alaska Native (AIAN), and Hawaiian and other Pacific Islanders applicants and matriculates have increased over the last fifteen years. However, they also found differences between racial groups, with Asian groups being over-represented and Black, Hispanic, and AIAN groups remaining underrepresented [8]. Another cross-sectional study estimated racial and ethnic diversity within the US healthcare workforce and healthcare graduates across 10 health professions [10]. In alignment, Salsberg and Colleagues [10] concluded that despite increasing numbers of Black, Hispanic, and Native American healthcare graduates entering the healthcare workforce, representation within the educational pipeline and workforce remained lower than the general working population [10]. These findings suggest that diversity initiatives need to improve strategies to retain racial and ethnic minorities within the health education and workforce pipeline. 

It has been suggested that implicit bias within health education settings contributes to challenges in recruiting and retaining future health professionals from racial and ethnic minorities and hampers diversification efforts [11,12]. Implicit racial bias refers to unconscious attitudes and beliefs towards a person based on their race that are outside of an individual’s awareness. In contrast, explicit racial bias refers to attitudes and beliefs towards an individual or group based on their race that are within the awareness of an individual and are associated with enacting behaviors with discriminatory intent [11,13]. Overt discriminatory behaviors are considered socially unacceptable and prohibited by law with many individuals expressing a belief in equality for all. However, several researchers have proposed that explicit and implicit bias persists in a subtle, pervasive manner [14].

Implicit bias is most frequently measured using the Implicit Association Test IAT [15], which is a computerized assessment of implicit cognition based on response latency. IATs can be used to quantify specific biases including racial, ethnicity, and skin tone bias. Participants are assigned a score based on reaction times i.e., the quicker concepts are sorted, the higher the unconscious association between those concepts. The IAT is now widely used to measure a range of biases, although it should be noted that several researchers have criticized the validity of this test [16,17]. Using the IAT as well as a range of survey and qualitative research methods, several studies have identified that various healthcare professionals and students hold implicit racial and skin tone bias [16,18,19]. 

### 1.1. The Rationale for the Present Review

Whilst research has examined implicit racial biases amongst healthcare professionals, the experiences and outcomes of experiencing implicit racial bias for healthcare students from racial and ethnic minority backgrounds remains sparse and thus, warrants attention for three reasons. First, implicit racial biases could have a deleterious impact on the mental wellbeing and academic performance of students from racial and ethnic minority groups [4,5,6,20,21]. For instance, Odom et al. [5] conducted a multisite focus group study of 43 minority medical students examining the facilitators and inhibitors of minority students’ personal and professional success in medical school. They found that racial and ethnic minority students described twice as many inhibitors than facilitators, including feeling ostracized amongst peers, perceptions that others considered them to be intellectually inferior, and that these experiences reduced their academic confidence. Moreover, a narrative review of 28 studies confirmed that students perceived their race to have a negative impact on their educational experience and satisfaction [4]. These findings are concerning given that research has reported that healthcare professional students have high rates of mental health conditions; for example, one-third of medical students report experiencing depression, and 10% experience suicidal ideation [22,23].

Second, the perpetuation of implicit racial bias in training materials and experiences during training could have a subsequent detrimental impact on the career development of qualified minority healthcare professionals [4,21,24]. For example, it has been suggested that minority medical students may be deterred from continuing with a career in academic medicine [4] or experience further challenges entering certain specialisms [25], particularly those with little workforce diversity due to lack of ethnic/racial representation [11]. For instance, while 12.4% of US Family Medicine/General Practitioners are ethnic minorities, only 0.8% of US Dermatologists are [26]. Racial and ethnic minority students may not pursue or consider specialisms with a lack of role models to avoid exposure to further negative experiences and feelings of isolation. This may counterproductively contribute to a further reduction of diversity within those medical specialisms and organizations, although further research to understand the reasons underlying these variations is needed.

Third, implicit racial biases are a key contributor to perpetuating health inequities in healthcare [18,19]. Shifting racial and ethnic demographics in local populations increases the need to be aware of different patient needs, cultural awareness, and competence within healthcare delivery. Several influential systematic reviews have shown that healthcare providers’ implicit racial bias is associated with lower quality patient-provider communication which can affect patient satisfaction and contribute to health disparities [18,19,27]. One study suggested that racial and ethnic composition of medical schools improved students’ self-reported preparedness to care for diverse patients, however, these effects were only observed in educational settings with positive interracial climates [28]. In order to retain an inclusive workforce, an improved understanding of the challenges and consequences of exposure to implicit racial bias within the learning environment is required. 

### 1.2. Aims and Context of the Present Review

While extensive evidence shows the wider implications of implicit racial bias on the care provided for racial and ethnic minority group patients [12], only a limited number of studies have explored providers’ experiences of implicit racial bias and there is even less research from the perspective of racial and ethnic minority healthcare students. This review aims to provide a narrative overview of the role of implicit racial bias in healthcare student education. More specifically, we will summarize and discuss evidence relating to trends, effects of implicit racial bias in healthcare student education, sources of bias, and interventions that address implicit racial bias.

The timing of this review is pertinent in the context of current social and political events. Inequalities witnessed during the ongoing coronavirus (COVID-19) pandemic (e.g., the higher mortality rate for racial and ethnic minority group patients and healthcare workers) and global outrage following the murder of George Floyd in 2020, have strengthened calls to address institutional inequalities. This has included calls for the diversification of the healthcare workforce to address health disparities, which has prompted a critical assessment of inclusivity within the current healthcare learning environment. Whilst many healthcare systems in the global north (or described in earlier terminology as ‘developed countries’) have a higher representation of certain racial and ethnic minority groups and international staff compared with the general population, they tend to be strongly over-represented at the lower pay bands and under-represented at the higher pay bands [29]. They are also more likely to experience harassment, bullying, and abuse whilst at work [30].

## 2. Materials and Methods

This narrative review identified relevant literature between May 2021–August 2021 through three main routes: (1) searches in three databases (EMBASE, CINAHL, and Ovid Medline) for original and peer-reviewed articles using specific search terms, ‘Healthcare students’, ‘racial bias’ and ‘healthcare’ within titles, abstracts, and keywords; (2) manual citation and hand searches for key articles were undertaken; and (3) searches in Google Scholar to ensure all recent and highly cited articles were included. According to Sukhera et al. [31], evidence for ‘implicit bias’ as an important aspect of human interaction was outlined in 1995, therefore searches were limited to articles published between 1995–2021. Studies were not included if they did not examine implicit bias in relation to race/ethnicity, non-healthcare-related topics, were not written in the English language or were unavailable in full text or grey literature. Relevant papers were collated using excel and grouped according to the main aims of this review. The focus and structure of the review were decided during discussions between researchers (O.R.J, JJ) based on the most concise format in which to present identified evidence.

## 3. Trends in Implicit Racial Bias Research in Healthcare Education

The development of the IAT by Greenwald and colleagues [15], and subsequent release of the US ‘Unequal Treatment’ (2003) report [32], strongly influenced a rise in implicit racial bias research in healthcare education. The report commissioned by the Institute of Medicine (IOM) highlighted bias, stereotypes, prejudice, and uncertainty as key factors contributing to persistent racial and ethnic health disparities and recommended healthcare providers receive cross-cultural education during training. 

The Unequal Treatment [32] report triggered a steady increase in healthcare education researchers examining the extent of racial bias and providing further evidence for healthcare organizations, educational institutions, and educators (clinical and non-clinical) to acknowledge and address their role in perpetuating disparities, and the need to raise awareness of the consequences of implicit racial biases earlier in healthcare training [33,34]. For example, research indicates that healthcare students and faculty have personal biases, but these are often not recognized [33]. Indeed, in a survey of one medical school, it was found that one in four students resisted recognition of personal biases [34]. Commentators have suggested that implicit racial biases may be a driver of poorer performance through additional burdens and stressors not experienced by white students [5], reduced access to resources and support [2], and increased exposure to negative stereotypes [35].

Despite the wide-ranging literature exploring implicit racial biases amongst qualified healthcare professionals, few studies have investigated how implicit racial biases affect student experiences within healthcare education settings. A recent review from the US examined implicit racial and ethnic bias in qualified and student healthcare providers using the IAT, but out of 37 studies, only 10 were in healthcare student populations. Of these 10 studies, 7 focused solely on medical schools and medical students whilst the remaining 3 sampled students in counseling, mental health trainees, and one combined pharmacy, nursing, and medical students [19]. Due to the lack of longitudinal research examining implicit racial bias in healthcare students, it is not possible to identify whether there have been changes in implicit bias in healthcare education settings over time or whether there are significant differences between countries. Studies of implicit racial bias in general populations indicate that implicit biases appear to be reducing overtime, and it is possible that this might be reflected in healthcare settings. However, further research is needed to clarify if this is true [36]. 

With regards to gaining a more nuanced understanding of the methods used to explore experiences of racial and ethnic minority group healthcare students compared with their white counterparts, common methods have included in-depth interviews [6] and focus groups [2,5], however, empirical evidence is sparse. Relevant experiences have been described in non-empirical articles including commentaries [37,38] and student perspective articles [21].

### Attainment Gap in Healthcare Education

Studies examining the attainment of racial and minority ethnic group students have mainly been conducted in the UK and US [1], with medical schools, investigated separately due to the pass/fail scoring system instead of degree classifications. Differential attainment by ethnicity refers to the average group difference in performance between white students and racial and ethnic minority group students and takes the students’ written and practical assessments into consideration. Some studies have found that racial and ethnic minority group students consistently underperform in comparison to white students and are significantly more likely to fail assessments [1,39,40]. 

Researchers have explored potential explanations for these differences, including racial/ethnic bias within standardized assessment processes such as examiner bias. One systematic review and meta-analysis of UK medical students and trainee doctors explored ethnicity and academic performance throughout the education and training pathway [1]. Woolf et al. [1] found a significant negative effect of ethnicity on academic performance, specifically that racial and ethnic minority medical students and trainee doctors were 2.5 times more likely to fail than their white peers. Furthermore, they analyzed differences between written and practical assessments which were machine or examiner marked to investigate examiner bias and verbal communication skills and found that the method of assessment was unlikely to be the main reason for performance differences between ethnic groups. Similarly, in another study, it was reported that that examiner bias did not influence scores or feedback assigned to racial and ethnic minority groups for Objective Structured Clinical Examination (OSCE) performances despite stereotype activation [41]. Finally, a recent critical review of 10 studies that analyzed OSCE examiners’ ethnicity and gender bias in healthcare education also corroborated that there was limited evidence to support bias propagated by individual assessors, the authors reported a lack of methodological quality of studies [42]. 

More recently, a retrospective analysis of written assessments for 1512 medical students in Scotland examined their performance at the end of Year 1 and end of Year 4 within the medical school, focusing on changes over time [3]. The findings indicated that non-white, international, and male students were in the top decile for academic performance before and at the end of their first year in medical school, which subsequently declined as they progressed, to non-white students being overrepresented in the bottom decile [3]. These studies suggest that factors other than academic ability may account for differential attainment. 

## 4. Sources of Implicit Racial Bias

Identifying the sources of implicit racial bias in healthcare education is vital to developing bias reduction interventions. In healthcare education settings, sources can be divided into four different groups relating to bias originating from peers, educators, placement environments, and the learning environments.

### 4.1. Peer to Peer 

Learning for healthcare students is a social process and involves individual and group work within both university and healthcare placements. As such, peer-to-peer relationships are important not only for student wellbeing but also for their academic and professional development. Indeed, many studies have shown that the majority of healthcare students have a white preference [43,44], positive bias towards light skin tones [45], and hold false negative beliefs about racial differences [46]. However, it is important to note that these studies only examined the role of implicit racial bias in relationships between providers and patients through vignettes, and there is only limited evidence of the impact of implicit racial bias in interpersonal interactions and relationships between students. As described previously, healthcare students’ implicit racial biases are in line with the general population and are either maintained or exacerbated during training [2,35,47], which could result in negative attitudes or behaviors towards their non-white peers. 

Gonzalez et al. [47] conducted 11 focus groups with 56 fourth-year medical students to explore student perceptions of racial bias teaching and students expressed that they would hesitate to share IAT results in a peer group discussion due to fear, shame and concerns for upsetting racial and ethnic peers. Moreover, a social network analysis of one UK medical school examined the relationship between the academic achievements of 158 years 3 and 4 medical students and ethnicity, role, and age homophily of learning groups. Homophily refers to a stronger social connection between individuals with similar characteristics. The findings revealed patterns of ethnicity and religious homophily in student networks, which affected their access to resources and support. However, racial and ethnic homophily had no impact on academic achievement [48]. These are similar to findings from a qualitative study, whereby medical and biomedical students described feeling excluded from social groups, feeling conscious of race during clinical placements, and experiencing an additional burden of appearing ‘professional’ [2]. The exact nature of implicit racial bias between peers in healthcare education settings is fragmented and unclear due to difficulties in differentiating unconscious habitual responses from subtle discrimination and modern forms of racism [49]. However, it can be inferred from the wider literature that implicit racial biases exist within these environments and could be present in interpersonal communication between peers [4,47,50]; also see commentaries, [37,38].

### 4.2. Healthcare Educators and Assessors

Qualitative studies examining student experiences have highlighted the lack of diverse faculty and assessors as a factor that adds to negative experiences faced by racial and ethnic minority group healthcare students. These studies report that racial and ethnic minority group healthcare students experience and witness racial prejudice and stereotyping from academic and clinical staff, adjust behavior to counteract negative stereotypes, and endure increased stress to prove intellectual ability [2,5,47,50]. For example, one minority student said ‘you have to be exceptional to be considered average’ [2] (p. 6). With regards to assessors, Claridge et al. [2] also found that clinical assessors lack understanding of how racial and ethnic minority students react to stressful situations, for example not realizing that they may not present with visible signs of anxiety such as blushing. 

An important finding is that clinical educators themselves do not feel proficient enough to deliver training about race and discrimination. Gonzalez et al. [51] conducted 21 in-depth interviews with faculty who had the experience of teaching implicit bias or had an interest in facilitating discussions and found that they felt they lacked the skills and knowledge to appropriately discuss bias especially when students were resistant to learning about bias or expressed strong emotive reactions which presents challenges within the learning environment.

In addition, healthcare educators’ personal biases have been shown to be enacted in various ways including lecture materials [24], clinical assessments [39], recommendation letters, and award systems [52,53]. For example, Lee et al. [52] found that racial and ethnic minority group students were marked down in clinical assessments and self-reported receiving more negative comments and fewer positive comments than white students. Another US study examined the word content of student performance evaluations and found an inconsistency in the words used to describe white students in comparison to racially diverse students [53]. These findings suggest that differential treatment during assessments could be a factor contributing to the ethnicity attainment gap.

### 4.3. Hospital and Clinic Environments during Placements

Three important studies have investigated implicit racial biases in relation to healthcare student placements. The first was in a cross-sectional study which examined the association between racial bias and attitudes towards psychiatric diagnosis (psychosis vs. mood disorder), ability to adhere to prescribed treatment (compliance vs. non-compliance), and choice of psychiatric medications (antipsychotics vs. antidepressants) in a sample of 122 medical students and 172 psychiatric physicians [54]. Londono Tobon et al. [54] found that self-reported white medical students and qualified psychiatric physicians were more likely to assign black faces to psychotic disorders, non-compliance, and antipsychotic medications. Furthermore, higher levels of training for the qualified healthcare professionals predicted an increased likelihood of moderate to strong assignment of black faces to psychotic disorders/antipsychotic words. 

The second study measured 3547 non-African American medical students’ changes in implicit racial bias from the first to the last semester of medical school and found that 48.7% of students heard negative comments about black patients from residents and senior staff during placements. Student exposure to these negative comments and poor experiences with African American physicians were both associated with a statistically significant increase in implicit racial bias [35]. However, the authors did not examine other residents’ and senior staffs’ comments regarding other racial groups during placements, therefore limited conclusions can be drawn. 

Finally, Claridge et al. [2] conducted interviews and focus groups with 8 staff and 41 students (biomedical science and medicine) at a UK university [24]. All students described experiences of perceived or anticipated prejudice and stereotyping by peers and university faculty, however, only black students reported racial prejudice during clinical placements. They emphasized increased stress and pressure to change their behavior to a Eurocentric view of a ‘professional’ to counteract anticipated negative stereotypes regarding behavior and appearance. One student stated ‘…just because I’m from South London and I’m Black, it doesn’t mean I’m going to be really rude or stand-offish. And I have to be very open and very nice and polite and avoid conflict with staff and with peers.’ [2] (p. 6). 

Together, these studies produce two key implications. First, unconscious patterns of bias in healthcare education settings are propagated by healthcare professionals in more senior roles. Second, these findings highlight how wider racial biases in healthcare settings could be experienced by and impact ethnic minority healthcare students resulting in disproportionate stress and pressure to their white peers. In particular, racial and ethnic minority healthcare students’ exposure to negative stereotypes associated with their racial group may contribute to feelings of isolation, and an added burden to not fulfill negative stereotypes described previously [5,6], which may contribute to poorer academic performance. However, it must be recognized racial and ethnic minorities are heterogeneous groups and future research should examine the difference between and within racial groups for differential effects of implicit racial bias. In addition, due to the geographical limitations of these studies (either conducted in the US or UK), it cannot be assumed that these findings and implications would translate to other settings.

### 4.4. Learning Environments

Many studies have evidenced that the learning environment influences healthcare students’ behaviors towards their peers and patients in clinical and education settings [4,5,55,56,57]. For example, a narrative review of the social and learning environments experienced by underrepresented racial and ethnic minority group medical students found they had perceptions of reduced social support, negative learning environments, increased levels of racial discrimination and harassment and attributed these factors to their race [4]. Despite over two decades of research, factors such as lack of diversity of people in leadership [9], lack of opportunities for skill and knowledge development for staff facilitating discussions about implicit racial bias, and racial bias in medical school admissions committees continue to persist [58]. The variable attitudes to the existence and impact of race-based biases in healthcare education expressed by leadership can contribute to this, due to power differentials and social influence of qualified healthcare professionals on impressionable healthcare students navigating what is considered acceptable. 

The formal, informal, and hidden curriculum continues to be a significant source of implicit racial bias in healthcare education settings. Informal norms communicated via the informal curriculum are simple and often positive, whereas the hidden curriculum refers to subtle messages during informal interactions, which can be positive or negative and relate to attitudes, beliefs, values, and behaviors, such as learning how to work with others. Calls to address the misrepresentation of race within pre-clinical healthcare education have increased in recent years from students, healthcare faculty, and healthcare professionals [20,21,24]. Within the formal curriculum, a range of studies has highlighted the need to reflect on the use of language when discussing or referring to race in the curriculum including lecture materials, exams, group discussions. For example, researchers analyzed approximately 350 pre-clinical lectures from a US medical school for racial signifiers and found that race was only mentioned in reference to biological risk or difference without context, explanation, or recognition of the social determinants. The authors suggested that the effect of this was to pathologize race and reinforce the negative racial stereotypes held by some students [24]. For example, amongst healthcare professionals, it is common to hear diseases associated with a racial group such as sickle cell and Black patients, however, the race is not the biological cause, rather the genetic mutations in populations within geographical areas at risk of malaria. Mediterranean individuals (who can be white-skinned) fall within this group, as well as individuals with mixed ancestry that may inherit the sickle cell allele and these racial assumptions based on physical characteristics can increase misdiagnosis [24,59]. Inadequate or incomplete discussions of race in healthcare curricula places undue importance on the biological difference between individuals without referring to the social factors that can contribute to determining health outcomes.

To date, only one study has investigated the impact of the hidden curriculum on implicit racial bias. This study conducted a longitudinal study of 2922 non-African American medical students across 49 US medical schools to explore the effect of formal and informal experiences on their attitudes toward African Americans [60]. It found that when non-African American students were exposed to disparaging racial remarks and jokes about black patients, they were significantly more likely to express racial bias [60]. These results suggest that the impact of the hidden curriculum may have a significant impact on healthcare professionals’ racial bias, but further studies in other countries and disciplinary groups are needed to explore this further. 

## 5. Personal Consequences of Racial Bias for Racial and Ethnic Minority Healthcare Students 

As discussed above, there is a significant attainment gap between white and minority racial and ethnic group students. This gap has previously been attributed to a lack of faculty and clinical staff diversity, the added burden of stereotypes and bias not experienced by white students, and a lack of personal and educational support from peers, faculty, and hospital staff [5,6]. Another contributing factor to differential academic attainment could be the complex interplay between students’ awareness of negative social stereotypes and prejudices related to their racial group and experiencing or witnessing implicit racial bias. Racialised healthcare students’ knowledge of negative stereotypes related to their racial group can increase the fear that their performance will be scrutinized or that they will fulfill negative stereotypes. This is an additional stressor called stereotype threat, where awareness of negative stereotypes associated with their racial group can increase responsibility to provide a counter-narrative, which in turn negatively affects academic performance [6]. 

Several reports from minority racial and ethnic group students describe experiencing ignorance and insensitive comments from fellow students, whereby faculty and clinical staff treated them as an ambassador or spokesperson for their entire race [2,5,6]. Moreover, an opinion piece written in the UK by two medical students referenced the increased burden of being regarded as a race representative and described the experience of being ‘black and female’ as a ‘double jeopardy’ [38]. Additionally, students highlight increased pressure to represent their racial group in the classrooms and on wards or are mistaken for a nurse during medical placements [5].

Increased stressors, additional ‘burdens’ of supporting equality diversity and inclusion initiatives, and perceptions of isolation are factors that contribute to negative experiences for racial and ethnic minority healthcare students. However, students also report positive experiences with racial and ethnic minority group patients during clinical placements, where patients appreciated interactions with racial and ethnic minority group healthcare professionals [5].

## 6. Current Interventions and Recommendations

It is important to consider which interventions may reduce racial and ethnic healthcare students’ exposure to implicit racial biases in healthcare education. It can be concluded from existing literature that common interventions involve educational activity, including raising awareness of the consequences of implicit bias, utilizing validated measures (e.g., IAT) to identify and address personal bias through discussion of results, facilitating interracial reflective spaces to acknowledge biases and training in bias mitigation strategies [11,56].

Despite, the development of diverse interventions, to recognize, reduce and manage implicit racial bias, only statistically small bias reduction effects have been observed [31,51]. For example, Stone and Colleagues [61] examined implicit stereotyping (association between Hispanic patient and non-compliance) in first-year medical students from majority (white), non-target minority (East Asian, Southeast Asian, or other non-White), and target minority (African American, Hispanic, or American Indian) groups. An IAT was conducted before and after two workshops (theory of implicit bias and an interactive session learning about reduction strategies to manage biases). They found a significant reduction of implicit stereotyping for majority groups, although the score suggests stereotyping of Hispanic patients and non-compliance was still apparent. It is also interesting to note, there was variance in the effectiveness of bias reduction between ethnic/racial groups, with non-target minority groups not showing any reduction in implicit stereotyping following the intervention. These authors recognized that further studies would benefit from utilizing longitudinal methods to explore whether bias reduction strategies can be sustained and how reduced stereotyping relates to clinical behaviors. Furthermore, interventions have mainly been explored within the US, and evidence of how different racial and ethnic groups respond to implicit racial bias interventions, warrant further investigation. To the authors’ knowledge, no interventions specifically focus on personal support for racial and ethnic minority healthcare students to improve their psychological and mental health. We will now discuss the main elements of each of these educational interventions below.

### 6.1. Raising Awareness of Implicit Racial Bias

Gonzalez and Colleagues have contributed substantial evidence to the implicit racial bias literature since 2014, seeking to understand barriers and facilitators to implicit bias instruction during healthcare education from a student, faculty, and institutional perspective. For example, one study invited third-year medical students to attend a single workshop about provider bias and health disparities, followed by a voluntary survey focusing on self-reported IAT results as well as their attitudes and experiences of the test and disparities in care provision. They were grouped as ‘deniers’ or ‘accepters’ depending on their response to the statement ’health disparities exist in the United States. Researchers found that 22% of participants doubted the validity of the IAT and these were the participants who were more likely to deny the statement [34]. Following these findings, these scholars have contributed further research to understand how to reduce student resistance to addressing personal biases and develop interventions to reduce racial bias with long-term effects [51,62,63]. 

Furthermore, they have tested theoretical and empirically informed strategies to improve teaching and learning experiences [34,47,51,56,62,63,64]. Gonzalez and Colleagues recently proposed 12 tips to reduce implicit bias; strategies include the PAUSE model, perspective taking, alternative narratives, critical reflection, and reflexivity [63]. These recommendations are focused at the individual level and complement strategies proposed by other authors [33,64], who highlight practical suggestions for institutions to lead on the reduction and management of race-based biases. On an institutional level, recommended interventions include developing a commitment to auditing current practices and processes to identify and eliminate biased language, ideology, and misrepresentations of race, allocation of adequate resources to build capacity amongst staff, enforcement of accountability, and implementing transparent reporting systems for students and faculty to report experiences of bias.

Some studies suggest that in addition to being encouraged to become more aware of their personal biases, healthcare students should be taught bias reduction strategies and that these should be framed appropriately to reduce self-blame and shame (e.g., highlighting professional development to be a good doctor rather than focusing on personal characteristics [11,63]. By incorporating implicit racial bias training across the curriculum, evidence shows increased acceptance and reduced resistance to race discourse and shows promise for the long-term reduction of implicit racial bias in healthcare [63]. 

### 6.2. Teaching Bias Mitigation Strategies 

Findings from previous research suggest that developing healthcare students’ skills and knowledge of implicit racial biases can instill an ongoing need for recognition and management of these unconscious thoughts and attitudes towards individuals considered as ‘other’ [11,63,64]. For example, Marcelin et al. [11] focus on two levels, individual and organizational. The individual-level emphasizes the need for continuous, structured critical reflection, and actively countering stereotypes through questioning and exposure to biased scenarios with appropriate responses (see [11] for the adapted Kirwan Institute framework). At the organizational level, the author proposed the development of an inclusion strategy to show commitment to reducing bias, from hiring and retaining diverse faculty to admissions and assessment committees. In addition, they suggest that leadership may benefit from an organizational mandate to combine the use of diversity training and the IAT to identify and address bias throughout the workforce. 

### 6.3. Reduce Misrepresentation of Race in the Healthcare Curriculum

Despite advances in our understanding of the complexities of racial and ethnic variation in humans, research indicates that misconceptions of race as finite biological categories continue to persist within healthcare education and practice [6,24]. Many scholars have proposed eliminating the reductive use of race/ethnicity categories within medical education by expanding discussions about the social determinants of health and the influences of social, political, and historic forces on an individuals’ health [33,64,65]. Evidence of the benefits of race, racism, and health disparities discourse is starting to emerge within single institutions. For example, in response to student activism, one medical education department created a task force with students and administration working together to redevelop the curricula. The multi-stage approach included asking first-year medical students to engage with materials about implicit bias (e.g., books and film), encouraging students to take the IAT to identify personal biases, and engaging in open discussions with peers and faculty. Additionally, skill and capacity development for faculty included a critical evaluation of race. Eight months later, the author reflected on the expansion of the acceptance of race and medicine in the school curriculum and culture [24]. However, approaches such as this are yet to be incorporated within the formal curricula nationally and internationally and future research may seek to measure outcomes to determine whether these approaches successfully reduce implicit racial bias amongst student populations. 

Another strategy that has been suggested involves the capacity development of faculty to deliver implicit racial bias instruction, developing their skills, knowledge, and confidence to educate on this highly emotive and polarizing topic regardless of their racial group [34,63]. In addition to this, researchers have proposed developing a standardized language of ‘race’ across the curriculum, which can reduce the hidden influence of those in higher positions of power exerting behaviors and attitudes that are inadvertently shaping healthcare students’ biases [24]. However, these strategies would involve substantial investment from organizations and have not been formally evaluated in research studies [56].

### 6.4. Organizational Commitment to Recruit and Retain Diverse Staff

One organizational approach to reducing implicit racial bias is to address recruitment policies and develop an inclusive environment that helps retain diverse staff. A recent article provides recommendations for institutions to consider when evaluating the influence of implicit racial bias on recruitment, retention, and advancement processes [66]. An example of the recommendations includes assessing the use of language in recruitment materials, considering where job opportunities are advertised, improving faculty reviewing processes to recognize talent within the diverse staff and provide clear information, mentoring, and support regarding formal processes for promotion (see [66], for all recommendations). Students recognize the need for diverse staff. Whitla et al. [67] reported that 83% of 639 medical students stated in a US telephone survey that diversity of the clinical faculty is important to enhance the educational experience. However, in a review study by Hernandez [68] it was proposed that if students are aware that staff has been appointed due to affirmative action policies, it reduces the effect of resistance to implicit racial bias instruction. Therefore, it is important to have a clear inclusion strategy and be transparent about institutional-level commitment to reducing bias in recruitment, hiring, and retention practices and processes [33].

### 6.5. Creating Trusting Spaces 

Commentators suggest that safe learning environments are vital to enable students to critically engage with race discourse and reduce the likelihood of self-guilt and shame that can lead to hesitant and resistant learners [47,69]. Some educational interventions draw on the sociological theory of intergroup contact which facilitates contact between members of different groups to increase positive attitudes between them. Whitla et al. [67] found that interracial contact with racially diverse peers led students from the dominant group to have increased knowledge, understanding, and awareness of equity issues. Many studies indicate the importance of informal networks and support groups and suggest faculty should help students to benefit from interracial communication by allocating diverse peers for group work. These connections can provide positive experiences that counter stereotypic presumptions, expand social networks, and increase access to knowledge and resources for racial and ethnic minority students that can determine success on the training course [48]. 

## 7. Future Directions

Both healthcare education and organizations are adopting various strategies to reduce implicit racial bias and improve racial and ethnic minority students’ experiences, performance, and retention. However, there are multiple sources of racial bias, and targets for intervention may need to explore combinations of strategies. The use of the IAT to measure implicit racial bias in healthcare student populations tends to focus on the impact of racial bias on clinical attitudes and behaviors towards diverse patients, with only limited high-quality empirical research exploring the impact of implicit racial biases on racial and ethnic minority healthcare students’ experiences and even less research outside of medical education settings. Much of the literature exists in the US and further research would benefit from insights across various geographical locations, larger sample sizes, and differential impact according to diversity within the student, faculty, clinical and local population, as this will determine exposure to interracial contact and interactions. 

Most educational interventions examining racial and ethnic minority healthcare student experiences tend to group all non-white racial groups as one homogenous group. Future research might explore differential experiences of healthcare education and placements across racial groups considered to be a minority. Many studies have highlighted the ethnicity differential attainment gap; however further evidence may benefit from qualitative approaches to develop an in-depth understanding of disparities between students from different racial groups. Finally, there is an urgent need for higher quality studies evaluating the effectiveness of interventions to reduce implicit bias in healthcare education settings. At present, few controlled studies are testing whether interventions are effective, and there is an overreliance on survey research. This is understandable; such studies would require organizational cooperation and commitment. However, this is crucial in order to enable understanding of which strategies are likely to be most effective and which should be supported more widely.

## 8. Limitations

The current study is a narrative review of the literature examining the experiences and impacts of implicit racial biases amongst healthcare students, and as noted further research is warranted to truly understand the wide-ranging implications. The approach is taken, a narrative review means that this study has identified previous research that describes the problem of interest. For a more comprehensive analysis, we suggest a systematic review to capture all research published on this topic. Nevertheless, this narrative review has highlighted the importance of understanding the impact of implicit racial bias on healthcare students, and the implication for healthcare professions if not addressed. This narrative review also highlights the need for further research exploration to identify effective interventions to address implicit racial biases and to prevent these experiences from occurring.

## 9. Conclusions

This review focused on understanding healthcare students’ experiences of implicit racial bias and found that students are exposed to racial biases within their learning and placement environments. Healthcare students are unintentionally exposed to biased attitudes, beliefs, and thoughts from fellow healthcare students, educators, and clinical staff, which can influence their verbal and non-verbal behavior towards racialized healthcare students leading to negative experiences such as isolation, reduced confidence, and lack of support. Much of the implicit racial bias literature focus on the downstream consequences for patient care delivery, however, further research may seek to explore how implicit racial prejudices, stereotypes, and attitudes affect relationships between healthcare students from diverse racial and ethnic backgrounds and how this impairs their social and learning experiences as well as their experiences within the clinical environment. Studies suggest that implicit racial bias is facilitated via different mechanisms and sources in healthcare, including students, faculty, healthcare professionals, as well as the curriculum and organizational culture, indicating that implicit bias is multifaceted in nature and likely to require multiple interventions. Several promising interventions have been developed and tested empirically, however the literature would benefit from larger sample sizes, multi-site studies for further understanding of how implicit racial bias negatively impacts healthcare student experiences. Further to this, few interventions address multiple sources of implicit racial bias and how they interact to lead to negative educational outcomes and experiences for underrepresented students, which can be difficult with such a nebulous concept. Future studies may usefully examine interventions with long-term reduction, investigate differential experiences between racialized students, and management of implicit racial bias.
